# Soluble Triggering Receptor Expressed on Myeloid Cells 1 Is Released in Patients with Stable Chronic Obstructive Pulmonary Disease

**DOI:** 10.1155/2007/52040

**Published:** 2007-11-05

**Authors:** Markus P. Radsak, Christian Taube, Philipp Haselmayer, Stefan Tenzer, Helmut R. Salih, Rainer Wiewrodt, Roland Buhl, Hansjörg Schild

**Affiliations:** ^1^Institute of Immunology, University of Mainz, Obere Zahlbacher Street 67, 55131 Mainz, Germany; ^2^III Medical Clinic, University of Mainz, Langenbeck Street 1, 55101 Mainz, Germany; ^3^University Medical Hospital, Department of Medicine II, University of Tübingen,Otfried-Müller-Street 10, 72076 Tübingen, Germany

## Abstract

Chronic obstructive pulmonary disease (COPD) is increasingly recognized as a systemic disease that is associated with increased serum levels of markers of systemic inflammation. The triggering receptor expressed on myeloid cells 1 (TREM-1) is a recently identified activating receptor on neutrophils, monocytes, and macrophage subsets. TREM-1 expression is upregulated by microbial products such as the toll-like receptor ligand lipoteichoic acid of Gram-positive or lipopolysaccharides of Gram-negative bacteria. In the present study, sera from 12 COPD patients (GOLD stages I–IV, ${\text{FEV}}_{1}$ 51 ± 6%) and 10 healthy individuals were retrospectively analyzed for soluble TREM-1 (sTREM-1) using a newly developed ELISA. In healthy subjects, sTREM-1 levels were low (median 0.25 ng/mL, range 0–5.9 ng/mL). In contrast, levels of sTREM-1 in sera of COPD patients were significantly increased (median 11.68 ng/mL, range 6.2–41.9 ng/mL, P<.05). Furthermore, serum levels of sTREM-1 showed a significant negative correlation with lung function impairment. In summary, serum concentrations of sTREM-1 are increased in patients with COPD. Prospective studies are warranted to evaluate the relevance of sTREM-1 as a potential marker of the disease in patients with COPD.

## 1. INTRODUCTION

Chronic obstructive pulmonary disease (COPD) is characterized by progressive development for the most part irreversible airflow obstruction that involves an abnormal airway inflammatory response [1]. The clinical course of COPD is typically dominated by intermittent exacerbations, responsible for the majority of the disease-associated morbidity and mortality [2, 3].

In recent years COPD has more and more been characterized as a systemic inflammatory disease. Several mediators have been found increased in blood of COPD patients indicating persistent systemic inflammation [4]. The origin of this inflammatory response is unknown but several different mechanisms including smoking itself, a cytokine “spill over” from the lungs, tissue hypoxia, and genetic factors [5] or persistent bacterial colonization of the airways [6–10] have been suggested.

The triggering receptor expressed on myeloid cells (TREM-1) is a recently identified activating receptor on neutrophil granulocytes (PMN), monocytes, and macrophage subsets [11, 12]. The expression of TREM-1 is upregulated by microbial products, that is, by toll-like receptor ligands such as lipoteichoic acid (LTA) of Gram-positive or lipopolysaccharide (LPS) of Gram-negative bacteria. Ligation of TREM-1 is synergistic with TLR agonists on the activation of receptor bearing cells for the release of inflammatory mediators like TNF-α and IL-8 and the initiation of neutrophil respiratory burst [11, 13]. We recently reported that a natural ligand for TREM-1 is present on platelets, although it still needs to be identified [14]. The biological significance of TREM-1 in acute inflammatory responses is documented in mouse models for septic shock, where competition of TREM-1 with a recombinant soluble TREM-1 fusion protein or an putative receptor blocking peptide derived from a conserved region of TREM-1 saved mice from lethal LPS challenge or bacterial sepsis [15–17].

TREM-1 is also produced in a soluble form [18] and released in humans after endotoxin exposition [19] or in patients suffering from severe pneumonia [20] or sepsis [21]. In these critically ill patients, elevated levels of soluble TREM-1 (sTREM-1) are detectable in bronchoalveolar lavage (BAL) fluid or in plasma, respectively, and have a high accuracy and sensitivity in detecting microbial infections as underlying disease [20, 22, 23]. In addition, the time course of sTREM-1 levels might be a useful parameter in predicting the outcome in sepsis patients [24, 25]. However, a limitation of these studies is certainly that only critically ill patients were examined. A recent study by Richeldi et al. demonstrates that an increase in sTREM-1 is also detectable in patients suffering from community acquired pneumonia caused by extracellular bacteria, but not in patients with interstitial lung disease or tuberculosis [26]. Furthermore, sTREM-1 has been associated with major abdominal surgery and peptic ulcer disease [27, 28].

In the present study, we developed a sensitive enzyme-linked immunosorbent assay (ELISA) that is able to detect pg/mL amounts of sTREM-1 in serum of patients. Using this new TREM-1 specific assay, we assessed the amount of sTREM-1 released in 12 patients suffering from COPD and 10 healthy individuals for sTREM-1 and indeed found elevated levels of sTREM-1 in patients COPD, which correlated with disease severity.

## 2. PATIENTS, MATERIALS, AND METHODS

### 2.1. Patients

Twelve patients with COPD, all current smokers or exsmokers, were recruited on the basis of their clinical diagnosis and lung function impairment. None of the patients had lung diseases other than COPD and all were in a stable clinical condition for at least 3 month. The control group comprised 10 healthy nonsmoking individuals without the sign of airway obstruction and other significant illness. The study was approved by the local Ethics Committee.

All patients with COPD were under treatment with inhaled $\beta_{2}$-adrenoceptor agonists and/or anticholinergics, 3 patients were treated additionally with inhaled steroids, 3 with systemic steroids, and 2 with additional theophyllin, whereas the healthy control subjects did not have medication. Regarding baseline characteristics, there were no other significant differences between groups, except for lung function (Table 1).

### 2.2. Assessment of lung function

Lung function measurements including the determination of forced expiratory volume in 1 s (FEV_1_), forced vital capacity (FVC), residual volume (RV), intrathoracic gas volume (ITGV), and single breath diffusion capacity for carbon monoxide (DLCO) were performed following established guidelines [29–31] using standard equipment (Masterlab, Jaeger, Höchberg, Germany). Bronchodilator responses were quantified as absolute and percent increase of FEV_1_ measured 15 minutes after inhalation of 200 μg salbutamol.

### 2.3. Transfectants

Full-length cDNA encoding TREM-1 cloned in the eukaryontic expression vector pcDNA3 (Invitrogen) were stably transfected in HEK293 cells with Fugene 6 (Roche) according to standard protocols. The TREM-1cDNAs have been described previously [11]. Transfectants were obtained after G418 selection.

The production recombinant TREM-1::IgG1 fusion protein has been described previously [14].

### 2.4. Monoclonal antibodies

TREM-1 specific monoclonal antibodies (clone 6B1) were raised by repeated immunization of BALB/c mice with a recombinant TREM-1::IgG1 fusion protein according to standard procedures. Hybridoma supernatants were first screened by ELISA against TREM-1::IgG1 and human IgG (Sigma-Aldrich, Taufkirchen), respectively. Supernatants reacting against TREM-1::IgG1, but not IgG, were subcloned twice and further screened by flow cytometry using TREM-1-transfected 293 cells. Antibodies (Abs) were purified by affinity chromatography on a protein G-Sepharose column. The mAb clones 1C5 and 6B1 were of isotype IgG1.

### 2.5. Detection of soluble TREM-1 by ELISA

For the detection of soluble TREM-1 (sTREM-1), anti-TREM-1 (6B1) mAb was coated at 0.5 μg/mL in PBS, then blocked by addition of 100 μL of 15% BSA for 2 hours at $37^{{}^{\circ}}\text{C}$ and washed. Afterward the standard (recombinant TREM-1::IgG1 in 7.5% BSA-PBS) and the samples were added and the plates were incubated for 2 hours at $37^{{}^{\circ}}\text{C}$. For analysis of patient samples, sera were diluted 1:10 in 5% BSA prior to addition to the plates. After incubation, plates were washed and the biotinylated detection polyclonal Ab anti-TREM-1 (R&D Systems) at 5 μg/mL in 7.5% BSA-PBS was added for 2 hours at $37^{{}^{\circ}}\text{C}$. Plates were then washed and streptavidine-HRP (1:8000 in 7.5% BSA-PBS) was added for 1 hour at $37^{{}^{\circ}}\text{C}$. Plates were then washed again and developed using the Tetramethylbenzidine Peroxidase Substrate System (KPL, Gaithersburg, Md). The absorbance was measured at 450 nm. Results are shown as means with SD of triplicates. The lower detection limit was defined by 2x SD of the blank values (5 pg/mL). The recovery rate was 90.6+/−1%. Intraassay variation was 7+/−1%, interassay variation was 6+/−5%.

### 2.6. Statistical procedures

Mean values, medians, and standard deviation (SD) were computed. sTREM-1 levels were compared between groups using Mann-Whitney-U test. Lung function and other values were compared between groups using unpaired t-test. Correlation analysis was performed by Spearman’s rank correlation. Statistical significance was assumed for P<.05.

## 3. RESULTS

### 3.1. Detection of sTREM-1 in serum by ELISA

**Figure 1:**
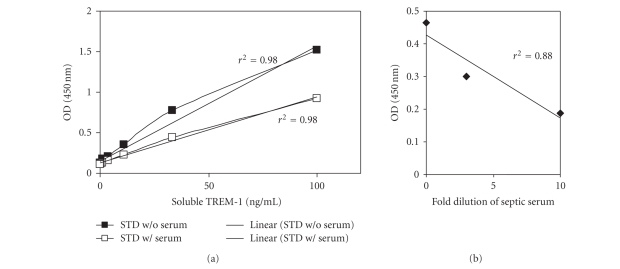
ELISA for sTREM-1. (a) Titration of recombinant human TREM-1::IgG1 either in the absence (filled symbols) or presence of normal human serum (open symbols). (b) Serial dilution of serum from a septic patient. The $r^{2}$ values are the correlation coefficients of the depicted data.

To evaluate the newly developed assay TREM-1-IgG recombinant human TREM-1::IgG1 and serum form a patient with sepsis were analyzed in serial dilutions since high levels of sTREM-1 have been described in sepsis previously [22]. As depicted in Figure 1, the assay allowed the detection of sTREM-1 down to 5 pg/mL sTREM-1 concluding that this new ELISA protocol is a suitable tool to investigate the significance of sTREM-1 in patients.

### 3.2. Serum levels of sTREM-1 are elevated in patients with COPD

**Figure 2:**
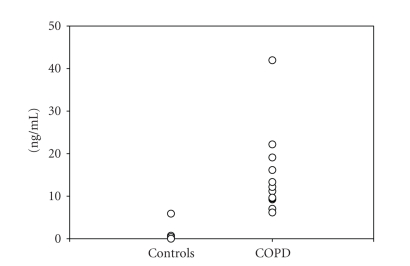
Concentration of sTREM-1 in serum of healthy controls (controls) and patients with COPD.

None of the control patients showed airway obstruction (Table 1), whereas COPD patients showed a significantly decreased FEV_1_ and FEV_1_% predicted (Table 1). According to GOLD criteria 2, patients were categorized as stage I (mild), 3 patients as stage II (moderate), 6 patients as stage III (severe) and 1 patients as stage IV (very severe) [32]. COPD patients also showed a significant increase in RV (P=.015) as well as ITGV (P=.035) and a significant decrease in DLCO (P<.001) compared to the control subjects (Table 1). None of the COPD patients showed a positive response to salbutamol, defined as an increase in FEV_1_ of at least 15% and 200 mL. Serum levels of sTREM-1 were significantly (P=.019) increased in patients with COPD compared to controls. In contrast, in healthy subjects sTREM-1 was detectable in serum samples of only 6 subjects (Figure 2).

### 3.3. Relationship between serum levels of sTREM-1 and clinical parameters

**Figure 3:**
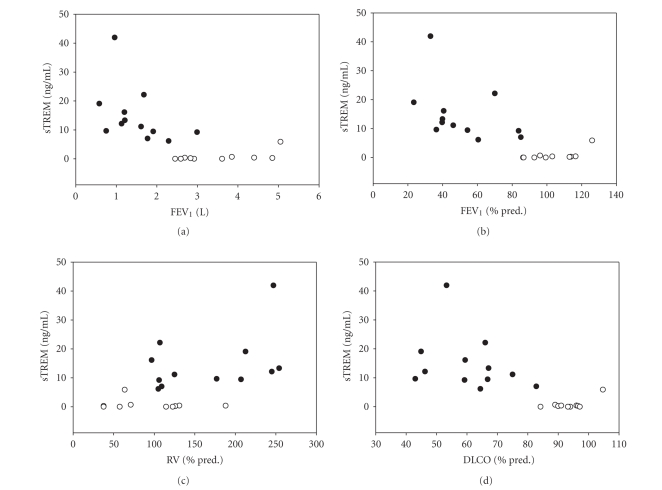
Relationship between sTREM-1 serum levels and absolute FEV_1_(panel A), FEV_1_% predicted (panel B), residual volume (RV) % predicted (panel C), and diffusion capacity (DLCO) % predicted (panel D). Control subjects are represented by open circles, patients with COPD by closed circles.

Levels of sTREM-1 in serum were correlated with absolute FEV_1_ (r=−0.74, P=.001), FEV_1_% predicted (r=−0.78, P<.001) (Figure 3) and FEV_1_% VC (r=−0.82, P<.001). Also significant correlations were detected to RV (r=0.48, P=.024), DLCO (r=−0.78, P<.001) and VC % predicted (r=−0.47, P=.028). No relationship was found between sTREM-1 and BMI (r=−0.28, P=.215), age of the patient (r=0.11, P=.64), height (r=−0.13, P=.553), or weight (r=−0.39, P=.069).

Due to the limited number of patients in our study, it was not possible to analyze correlations between lung function parameter and sTREM-1 levels.

## 4. DISCUSSION

In the present study, we show that sTREM-1 levels in serum are elevated in patients with COPD compared to healthy, nonsmoking controls. Furthermore, we demonstrate that serum levels of sTREM-1 are correlated with disease severity.

COPD is a multicomponent disease which includes inflammatory changes in the lung. Inflammatory cells in lungs of patients with COPD are mainly neutrophil granulocytes and inflammation in the lung is more pronounced with worse lung function [33]. Also, numbers of neutrophils in sputum correlate with disease progression [34]. In many patients, COPD has also significant systemic consequences. This includes loss of lean bodymass, cardiovascular effects, osteoporosis, muscle wasting but also a systemic inflammatory response. Indeed, in patients with COPD, even during stable disease, there is an increased number of leukocytes in peripheral blood [35, 36] Peripheral neutrophils from patients with COPD show enhanced chemotaxis and extracellular proteolysis [37], produce more reactive oxygen species [38], and enhanced expression of several surface adhesion molecules [39]. Also increased levels of TNF-α, IL-6, IL-8, C-reactive protein, and fibrinogen in serum can be detected in serum of patients with stable COPD and some of these systemic inflammatory changes seem to be related to disease severity [4] and correlate with severity of systemic consequences like muscle wasting [40].

In the present study, we describe a new ELISA for the detection of sTREM-1 in serum. In contrast, most previous studies have used an immunodot blot technique for the detection of sTREM-1 [22, 25, 41]. This technique allows the sensitive detection of sTREM-1 in body fluids. However, for assessment of sTREM-1 in a clinical routine setting detection by sandwich ELISA has the advantage that greater sample numbers may be processed simultaneously with increased specificity. Therefore, we developed a new sensitive ELISA using a monoclonal antibody to capture sTREM-1 in sera of patients and a polyclonal anti-TREM-1 for detection. Using our new sandwich-ELISA protocol, we established a standard laboratory method for the sensitive and specific detection of sTREM-1 in body fluids avoiding the pitfalls and potential disadvantages associated with the immunodot blot technique used so far. When analyzing serum samples from patients with stable COPD, we were able to detect sTREM-1 in all samples. In contrast, in samples from healthy control subjects sTREM-1 was only detectable in 6 of the patients and only in very low amounts. In line with previous data obtained from patients suffering from severe inflammatory disorders like pneumonia or sepsis [22–24], our data indicate that sTREM-1 might be a result of neutrophil activation also in COPD patients. The current view in terms of the pathophysiology suggests that sTREM-1 is an anti-inflammatory mediator of sepsis [42], released as a counter regulator of TREM-1 mediated activation. Our recent results demonstrating that sTREM-1 interferes with the TREM-1/ligand interaction of neutrophil and platelets support this view [14].

Previous studies have described increased levels of sTREM-1 in patients with sepsis [22], pneumonia [20] but also exacerbated asthma and COPD. By using an immunoblot technique, Phua et al. found increased levels of sTREM-1 especially in patients with COPD during an Anthonisen-type 1 exacerbation [41]. 

The authors speculated that the patients with type 1 exacerbation shad higher airway bacterial loads, which triggered systemic inflammation and increased sTREM-1 levels [43, 44]. In the present study, increased levels of sTREM-1, measured by ELISA, were detected even in patients with stable disease. This could be due to an increased sensitivity of the newly introduced ELISA.

Potential explanations for the increased levels of sTREM-1 observed in this group of stable patients with COPD could be persistent bacterial colonization of the airways, which has been demonstrated by several groups [6–9]. These colonizations can be associated with elevated levels of inflammatory mediators like IL-8, LTB4, and TNF-α in the lungs of patients with clinically stable disease [10]. In addition, increased plasma fibrinogen and IL-6 levels are detectable in these bacterially colonized patients suggesting that colonization contributes to systemic inflammation [44]. In patients with more advanced disease, higher levels of serum CRP have been described, suggesting increased systemic inflammatory reaction [45–47]. As sTREM-1 can be induced by increased systemic inflammation [20, 23, 24] the increased sensitivity of our ELISA assay could be the reason for increased levels of sTREM-1 detected in the present study population.

Additionally, we found a correlation of sTREM-1 levels in serum with disease severity, described by impaired lung function, in the COPD group. This was the case when absolute and relative values of FEV_1_ as markers for airway obstruction, RV for hyperinflation, and also diffusion capacity as a marker for emphysema were analyzed. No correlations were found for age, height, weight, or BMI. Also levels of sTREM-1 in serum were independent for medication used by patients. Some of the patients with more severe disease (GOLD stadium III–IV) treated with systemic steroid still showed increased levels of sTREM-1 in serum. Although this analysis is somewhat limited by low numbers of patients and control sTREM-1 may yet be another systemic marker correlated with disease severity.

In summary we show increased serum levels of sTREM-1 in patients with clinical stable COPD, and a correlation between serum levels and disease severity. sTREM-1 might be a useful marker for systemic inflammation in patients with COPD. Further prospective studies are needed to evaluate the true diagnostic value of sTREM-1 in this patient population.

## Figures and Tables

**Table 1:** Patients’ characteristics ( ND not done).

	Control	COPD
Sex (f/m)	4/6	5/7
Age (y)	51±14	56±10
Height (cm)	171±9	169±8
BMI ($\text{kg}/{\text{m}}^{2}$)	26.9±6.1	23.3±4.5
Pack years of smoking	0	$30\pm 12^{*}$
FEV_1_ (L)	3.52±0.97	$1.51\pm 0.68^{*}$
FEV_1_ (%predicted)	103±13	$51\pm 20^{*}$
FEV_1_/FVC (%)	73±4	$52\pm 10^{*}$
ΔFEV_1_ Salbutamol (%)	N.D.	3.8±1.6
RV (%predicted)	95±49	$165\pm 63^{*}$
ITGV (L)	3.57±1.6	$4.32\pm 1.4^{*}$
DL_CO_ (%predicted)	94±6	$61\pm 12^{*}$
sTREM-1 (ng/mL)	0.25 (0–5.9)	11.68 (6.2–41.9${)}^{*}$

* For abbreviations, see text. Mean values ± SD are given. For sTREM-1 median and range are given P<.05* regarding the comparison between groups.
